# Increased Excitability and Heightened Magnitude of Long-Term Potentiation at Hippocampal CA3–CA1 Synapses in a Mouse Model of Neonatal Hyperoxia Exposure

**DOI:** 10.3389/fnsyn.2020.609903

**Published:** 2021-01-06

**Authors:** Manimaran Ramani, Kiara Miller, Namasivayam Ambalavanan, Lori L. McMahon

**Affiliations:** ^1^Department of Pediatrics, The University of Alabama at Birmingham, Birmingham, AL, United States; ^2^Departments of Cell, Developmental, and Integrative Biology, The University of Alabama at Birmingham, Birmingham, AL, United States

**Keywords:** early-life insult, hyperoxia, preterm, long-term hippocampal dysfunction, synaptic transmission, neuronal excitability

## Abstract

Preterm infants exposed to supraphysiological oxygen (hyperoxia) during the neonatal period have hippocampal atrophy and cognitive dysfunction later in childhood and as adolescents. Previously, we reported that 14-week-old adult mice exposed to hyperoxia as newborns had spatial memory deficits and hippocampal shrinkage, findings that mirror those of human adolescents who were born preterm. The area CA1 region of the hippocampus that is crucial for spatial learning and memory is highly vulnerable to oxidative stress. In this study, we investigated the long-term impact of neonatal hyperoxia exposure on hippocampal CA3–CA1 synaptic function. Male and female C57BL/6J mouse pups were continuously exposed to either 85% normobaric oxygen or air between postnatal days 2–14. Hippocampal slice electrophysiology at CA3–CA1 synapses was then performed at 14 weeks of age. We observed that hyperoxia exposed mice have heightened strength of basal synaptic transmission measured in input-output curves, increased fiber volley amplitude indicating increased axonal excitability, and heightened LTP magnitude at CA3–CA1 synapses, likely a consequence of increased postsynaptic depolarization during tetanus. These data demonstrate that supraphysiological oxygen exposure during the critical neonatal developmental period leads to pathologically heightened CA3–CA1 synaptic function during early adulthood which may contribute to hippocampal shrinkage and learning and memory deficits we previously reported. Furthermore, these results will help shed light on the consequences of hyperoxia exposure on the development of hippocampal synaptic circuit abnormalities that could be contributing to cognitive deficits in children born preterm.

## Introduction

Children and adolescents who were born very preterm are at higher risk of a lower intelligence quotient (Brydges et al., 2018) and deficits in executive function and processing speed (Lundequist et al., 2015; Brydges et al., 2018). They are also at high risk for developing attention deficit hyperactivity disorder and autism spectrum disorder (Rommel et al., 2017; Franz et al., 2018) than their counterparts who were born at term. In the neonatal intensive care unit, oxygen (O_2_) is the most widely used therapy. Extremely preterm infants (gestational age ≤28 weeks) frequently require prolonged periods of high concentrations of O_2_ due to the immaturity of their lungs. Under normal conditions *in utero*, with greater adaptive mechanisms allow an adequate supply of O_2_ to the tissues, the fetus develops in a relatively hypoxemic environment (fetal oxygen saturation of 80%; Castillo et al., 2008). In contrast, for *ex-utero* preterm infants, organ development occurs in a relatively hyperoxemic condition as the targeted range for O_2_ saturation (saturation range 88–95%) is higher than fetal levels, which could have a deleterious effect on organ development (Askie et al., 2017). The high concentrations of unsaturated fatty acids, high rate of O_2_ consumption, and low concentrations of endogenous antioxidants make the developing brain highly vulnerable to oxidative stress (OS; Ikonomidou and Kaindl, 2011). Though cumulative OS has been linked with neurodegenerative disorders such as Alzheimer’s disease (Cioffi et al., 2019) and Parkinson’s disease (Crotty et al., 2017), the impact of OS during the critical developmental period on brain development and function later in life is not well known.

Previously, we have shown that young adult C57BL/6J (14-week-old) mice that are exposed to 85% O_2_ (hyperoxia) from postnatal day (P) 2–14 (neonatal) exhibit deficits in spatial learning, show signs of hyperactivity and have shrinkage of area CA1 of the hippocampus (Ramani et al., 2013), a brain region central to normal learning and memory (Squire, 1992; Tsien et al., 1996; Squire, 2004; Eichenbaum, 2017; Stevenson et al., 2018) that is highly vulnerable to OS (Wilde et al., 1997; Wang et al., 2007; Wang and Michaelis, 2010; Medvedeva et al., 2017). Recently, we have shown that hyperoxia exposure during the neonatal period (P2–14) permanently impairs hippocampal mitochondrial function, increases proton leak in the mitochondria, and alters complex I enzyme function when assessed at young adult age (Ramani et al., 2019). Adequate mitochondrial function is essential for normal synaptic function and long-term plasticity as well as the formation and maintenance of learning and memory. An altered hippocampal mitochondrial function could increase neuronal reactive oxygen species (ROS), disturb homeostasis, and lead to abnormalities in the induction and maintenance of hippocampal long-term potentiation (LTP), a form of long-term synaptic plasticity which results in a persistent strengthening of synaptic transmission, contributing to long-term memory formation and maintenance (McGaugh, 2000; Levy et al., 2003; Cheng et al., 2010; Todorova and Blokland, 2017; Asok et al., 2019; Rossi and Pekkurnaz, 2019). While our previous research has shown that early life oxidative stress leads to deficits in cognitive-behavioral performance measured at 14 weeks of age, which are likely permanent, the mechanisms by which this occurs are still unknown.

In this study, we hypothesized that prolonged exposure to hyperoxia during the neonatal period would alter CA3–CA1 hippocampal synaptic function when assessed in early adulthood.

## Materials and Methods

All protocols were approved by the UAB Institutional Animal Care and Use Committee (IACUC) and were consistent with the PHS Policy on Humane Care and Use of Laboratory Animals (Office of Laboratory Animal Welfare, Aug 2002) and the Guide for the Care and Use of Laboratory Animals (National Research Council, National Academy Press, 1996). Unless denoted, all experiments were done with at least five to six mice of either sex from at least two litters for each experimental condition.

### Animal Model

C57BL/6J dams and their pups were exposed to either normobaric hyperoxia (85% O_2_) or normobaric 21% O_2_ ambient air (Air) from second postnatal day (P2) until 14 days of age (P14; Ross et al., 2006; James et al., 2010). Dams were alternated every 24 h from hyperoxia to air to reduce hyperoxia-induced adult lung injury. After the 14th postnatal day (P14), mice were returned to air and maintained on a standard rodent diet and light/dark cycling in micro isolator cages until assessment at 14 weeks of age. A series of electrophysiological assessments were performed to study basal synaptic transmission, neurotransmitter release probability, and the induction of LTP. Animals of both sexes were used in this study.

### Hippocampal Slice Preparation

Animals were deeply anesthetized *via* inhalation of isoflurane, rapidly decapitated, and brains were removed. Coronal sections (350 μm) from the dorsal hippocampus were prepared using a vibratome (Leica VT10000A). To preserve neuronal health and limit excitotoxicity, slices were sectioned in low Na^+^, sucrose-substituted ice-cold artificial cerebrospinal fluid [aCSF; in mM: NaCl 85; KCl 2.5; MgSO_4_ 4; CaCl_2_ 0.5; NaH_2_PO_4_ 1.25; NaHCO_3_ 25; glucose 25; sucrose 75 (saturated with 95% O_2_, 5% CO_2_, pH 7.4)]. Slices were held at room temperature for 1 h in aCSF [in mM: 119.0 NaCl, 2.5 KCl, 1.3 MgSO_4_, 2.5 CaCl_2_, 1.0 NaH_2_PO_4_, 26.0 NaHCO_3_, 11.0 Glucose (saturated with 95% O_2_, 5% CO_2_, pH 7.4)] before transfer to bath submersion chamber warmed to 26–28°C for recordings.

### Electrophysiology

As previously described (Smith and McMahon, 2018b), extracellular field excitatory postsynaptic potentials (fEPSPs) were recorded from the dendrites of CA1 pyramidal cells. CA3 axons were stimulated using a stainless-steel electrode (FHC, Bowdoin, ME, USA) placed in the middle of the CA1 stratum radiatum between the CA1 pyramidal cell layer and the hippocampal fissure within 200–300 μm of an aCSF-filled glass recording electrode, also within CA1 stratum radiatum. Baseline fEPSPs were obtained by delivering a 0.1 Hz stimulation for 200 μs to generate fEPSPs of ~0.5 mV in amplitude (~50% of maximal response). The paired-pulse facilitation (PPF) characteristic of this synapse was elicited using an inter-stimulus interval of 50 ms (Wu and Saggau, 1994). All data were obtained using pClamp10 software (Molecular Devices, LLC, San Jose, CA, USA) and analyzed using GraphPad Prism 7 (GraphPad Software, Incorporation, and SigmaPlot V12 (Systat Software, Incorporation). Only experiments with ≤8% baseline variance were included in the final data sets.

Input/Output curves: following a stable 10-min baseline, input-output (I/O) curves were generated by increasing the stimulus intensity (10 μA increments) until a maximal fEPSP slope was obtained. The initial slopes of the six fEPSPs generated at each stimulus intensity were averaged and plotted as a single value. Fiber volley amplitudes were also measured to determine the change in axonal activation across the same stimulation range (0–320 μA). Statistical significance was determined using a two-tailed and unpaired Student’s *t-*test at the maximal stimulus intensity (320 μA; *p < 0.05).

Paired-Pulse Ratio (PPR): PPR was determined by dividing the fEPSP slope of the second event in a pair of pulses at 50 ms intervals by the slope of the first fEPSP. Statistical significance was determined using a two-tailed unpaired Student’s *t*-test at the maximal stimulus intensity (320 μA; *p < 0.05).

Long-term potentiation (LTP): following a 20 min stable baseline (0.1 Hz, 200 μs duration with the stimulus strength set to elicit initial fEPSP amplitude of ~50% maximum response), LTP was induced using one bout of theta-burst stimulation (TBS). Each bout of TBS consists of five pulses at 100 Hz repeated 10 times at 200 ms intervals (weak TBS). Weak TBS was chosen to ensure that differences in the LTP magnitude were not masked by using a strong TBS that could induce maximal LTP. Statistical significance was determined using a two-tailed unpaired Student’s *t*-test comparing the average of the fEPSP slope from the last 5 min of the recording (44–59 min) to baseline for the air-exposed and hyperoxia-exposed groups (*p < 0.05, **p < 0.01).

Postsynaptic depolarization to induce LTP: to determine if TBS generated a different amount of postsynaptic depolarization between experimental groups during LTP induction, the area under the curve (AUC) was measured at 100 ms from baseline. This arbitrary time point was chosen due to the combined influence of NMDAR and AMPAR-mediated currents being the most impactful. Statistical significance was determined using a two-tailed unpaired Student’s*t*-test (*p < 0.05).

Coastline Burst Index: as previously described (Stewart et al., 2017), CA1 dendritic excitability was quantified using a modified version of the Coastline Burst Index (mCBI; Korn et al., 1987). The equation, ∑_25^125_(|*V*_x+1_ − *V*_x_|), was used to calculate mCBI, which summates the voltage differential between each point in the waveform (sampled at 10k Hz) beginning 5 ms after the stimulus artifact (25 ms). The equation also encompasses all repetitive population spikes activities (25–70 ms corresponding to 2,000 total data points per waveform).

### Statistical Analysis

The experimenter was blind to the groups, data collection, and analysis. Results were expressed as means ± SE with significance set at *p* < 0.05 (*) determined by two-tailed unpaired Student’s *t*-test assuming unequal variance comparing results from air to hyperoxia exposed mice. Sufficient power was determined with G*Power 3.1.9.2 (Franz Faul, University Kiel, Germany). Outliers were determined with a Grubb’s test (GraphPad Software, Incorporation), and significant outliers were removed.

## Results

### Basal Synaptic Transmission Is Significantly Altered at CA3–CA1 Synapses in Hyperoxia-Exposed Mice

**Figure 1:**
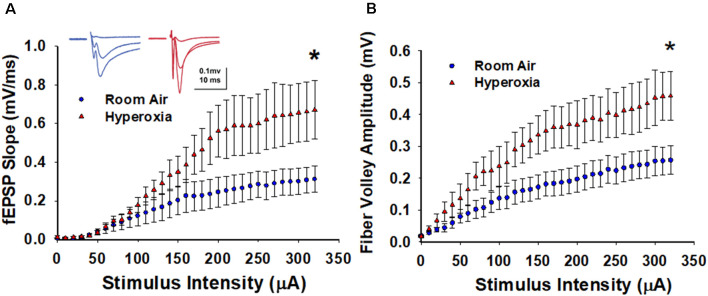
Long-term effect of neonatal hyperoxia exposure on CA3–CA1 synapses basal synaptic transmission. **(A)** Input-Output curves. Blue circles represent the air-exposed group, and red triangles represent the hyperoxia-exposed group. The maximum field excitatory postsynaptic potentials (fEPSP) slope is significantly larger at CA3–CA1 synapses from the young adult mice exposed to neonatal hyperoxia compared to air-exposed mice. Unpaired Student’s *t*-test at 320 μA means ± SEM; Air = 0.31 ± 0.06, Hyperoxia = 0.67 ± 0.15, *p* = 0.04, *n* = 6 slices/6 animals in Air, and 6 slices/6 animals in 85% O_2_. *Represents *p* < 0.05; Air vs. Hyperoxia. **(B)** Fiber volley amplitude. Blue circles represent the air-exposed group, and red triangles represent the hyperoxia-exposed group. Compared to air-exposed mice, fiber volley amplitude at the CA3–CA1 synapse was significantly larger in the young adult mice exposed to neonatal hyperoxia. Unpaired Student’s *t*-test at 320 μA; mean ± SE; Air = 0.75 ± 0.19, Hyperoxia = 1.44 ± 0.23, *p* = 0.04, *n* = 6 slices/6 animals in Air, and 6 slices/6 animals in 85% O_2_. *Represents *p* < 0.05; Air vs. Hyperoxia.

To determine the lasting impact of early life O_2_ exposure on the strength of excitatory input from CA3 pyramidal cells onto CA1 pyramidal cells, we measured input-output (I/O) curves generated by incrementally increasing the stimulus intensity (0–320 μA, 10 μA intervals). As shown in Figure 1A, the maximum fEPSP slope is significantly larger at CA3–CA1 synapses measured in slices from the young adult mice exposed to neonatal hyperoxia compared to air-exposed mice (Figure 1A, at 320 μA, mean ± SE; Air = 0.31 ± 0.06, Hyperoxia = 0.67 ± 0.15, *p* = 0.04, *n* = 6 slices/6 animals in Air, and 6 slices/6 animals in Hyperoxia). To determine if this increase in basal synaptic strength might result from an increase in presynaptic excitability, we re-analyzed the data and plotted fiber volley amplitude, a measure of axon depolarization, vs. stimulus strength. Again, we found that the maximum fiber volley amplitude at CA3–CA1 synapses was significantly larger in young adult mice exposed to neonatal hyperoxia (Figure 1B, at 320 μA, mean ± SE; Air = 0.25 ± 0.04, Hyperoxia = 0.45 ± 0.07, *p* = 0.03, *n* = 6 slices/6 animals in Air, and 6 slices/6 animals in Hyperoxia). Together, these data support the interpretation that early life O_2_ exposure increases presynaptic excitability resulting in heightened basal strength at the CA3–CA1 synapses.

### The Paired-Pulse Ratio Is Not Altered in CA3–CA1 Synapses in Young Adult Mice Exposed to Neonatal Hyperoxia

**Figure 2:**
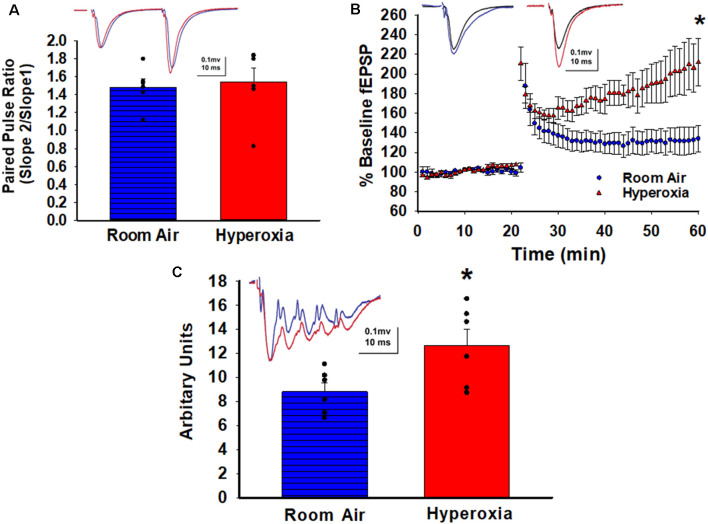
Long-term effect of neonatal hyperoxia on presynaptic probability and the magnitude of long-term potentiation in young adult mice. **(A)** Paired Pulse Ratio (PPR). A blue bar with horizontal lines represents the air-exposed group. The solid red bar represents the hyperoxia-exposed group. No differences in the PPR were detectable between the adult mice that had either neonatal hyperoxia or air exposure at the CA3–CA1 synapse. Unpaired Student’s *t*-test mean ± SE; Air = 1.48 ± 0.08, Hyperoxia = 1.54 ± 0.15, *p* = 0.75, *n* = 5 slices/5 animals in Air, and 6 slices/6 animals in 85% O_2_. **(B)** Long-Term Potentiation (LTP). Blue circles represent the air-exposed group, and red triangles represent the hyperoxia-exposed group. The magnitude of theta-burst stimulation (TBS) induced LTP was significantly greater at the CA3–CA1 synapses in the young adult mice exposed to neonatal hyperoxia compared to air-exposed mice. Unpaired Student’s *t*-test: means ± SEM%; Air-exposed: 134 ± 12% of baseline fEPSP slope vs. Hyperoxia-exposed 217 ± 23% of baseline fEPSP slope, *n* = 5 slices/5 animals in Air, and 6 slices/6 animals in 85% O_2_. *Represents *p* < 0.01; Air vs. Hyperoxia. **(C)** Maximum depolarization at tetanus. A blue bar with horizontal lines represents the air-exposed group. The solid red bar represents the hyperoxia-exposed group. Young adult mice that had hyperoxia exposure as neonates had a greater magnitude of the postsynaptic depolarization during the TBS. Unpaired Student’s *t*-test: mean ± SE; Air = 8.81 ± 0.73, Hyperoxia = 12.66 ± 1.34, *p* = 0.03, *n* = 5 slices/5 animals in Air, and 6 slices/6 animals in 85% O_2_.^ *^Represents *p* < 0.05; Air vs. Hyperoxia.

To determine whether early-life O_2_ exposure alters the presynaptic release probability, we analyzed PPR, an indirect measure of presynaptic neurotransmitter release probability (Dobrunz et al., 1997). No differences in the PPR at CA3–CA1 synapses were detectable between the adult mice that had either neonatal hyperoxia or air exposure (Figure 2A; mean ± SE; Air = 1.48 ± 0.08, Hyperoxia = 1.54 ± 0.15, *p* = 0.75, *n* = 5 slices/5 animals in Air, and 6 slices/6 animals in Hyperoxia), suggesting that presynaptic release probability in adulthood is likely unaffected.

### Heightened LTP Magnitude at CA3–CA1 Synapses in Young Adult Mice Exposed to Neonatal Hyperoxia

Next, we asked whether the increased excitability observed in the input/output curves would lead to heighten LTP magnitude since strong depolarization facilitates the removal of the voltage-dependent Mg^2+^ block from NMDARs. Indeed, a weak theta-burst stimulation (TBS; one bout of TBS with each bout consisting of five pulses at 100 Hz repeated 10 times at 200 ms interval) induced a greater magnitude of LTP at CA3–CA1 synapses measured at 44–59 min post-TBS in young adult mice exposed to hyperoxia compared to air-exposed young adult mice (Figure 2B; Air-exposed: 134 ± 12% of baseline fEPSP slope vs. Hyperoxia-exposed 217 ± 23% of baseline fEPSP slope, **p < 0.01, *n* = 5 slices/5 animals in Air, and 6 slices/6 animals in Hyperoxia). The greater LTP magnitude was not a consequence of stronger stimulation during baseline since the statistical comparison of baseline fiber volley amplitudes were not different between groups (Air: 0.27 ± 0.05 mV vs. Hyperoxia: 0.18 ± 0.09 mV, *p* = 0.42). Next, when we analyzed the magnitude of the postsynaptic depolarization during the TBS, we found a larger depolarization in young adult mice exposed to hyperoxia during the neonatal period compared to air-exposed mice (Figure 2C; mean ± SE; Air = 8.81 ± 0.73, Hyperoxia = 12.66 ± 1.34, *p* = 0.03, *n* = 5 slices/5 animals in Air, and 6 slices/6 animals in Hyperoxia), which could explain the greater magnitude LTP in hyperoxia-exposed mice.

### Coastline Burst Index Indicates Hyperexcitability

**Figure 3:**
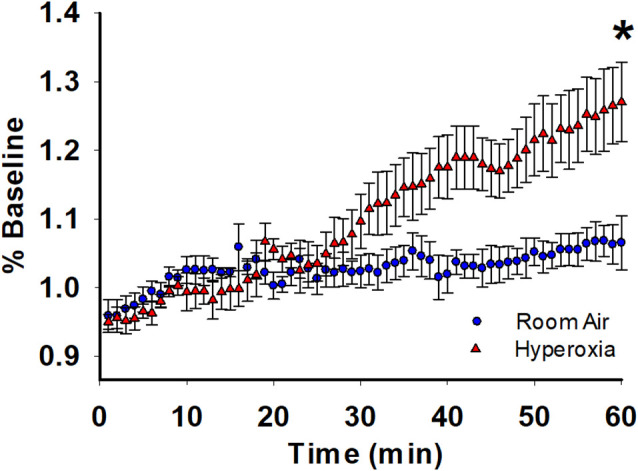
Long-term effect of neonatal hyperoxia on CA3–CA1 synapse excitability in young adult mice. Blue circles represent the air-exposed group, and red triangles represent the hyperoxia-exposed group. Young adult mice exposed to neonatal hyperoxia had a higher mean coastal burst index compared to air-exposed mice. Unpaired Student’s *t*-test: mean ± SE; Air = 1.06 ± 0.03, Hyperoxia = 1.27 ± 0.05, *p* = 0.02. *n* = 5 slices/5 animals in Air, and 6 slices/6 animals in 85% O_2_. *Represents *p* < 0.05; Air vs. Hyperoxia.

For further analysis of the depolarization during TBS, we used coastline burst analysis as a measure of heightened excitability. We found that young adult mice exposed to neonatal hyperoxia had a higher mean CBI percentage compared to room-air exposed mice (Figure 3, mean ± SE; Air = 1.06 ± 0.03, hyperoxia = 1.27 ± 0.05, *p* = 0.02, *n* = 6 slices/6 animals in Air, and 6 slices/6 animals in Hyperoxia). Collectively these data are consistent with the interpretation that the CA1 dendrites in young adult mice exposed to hyperoxia during the neonatal period are hyperexcitable, perhaps a consequence of increased presynaptic excitability (Figure 1B) compared to air-exposed young adult mice.

## Discussion

This is the first preclinical study to investigate the consequence of early life hyperoxia exposure, a condition experienced by preterm infants, on hippocampal synaptic function and long-term plasticity. Using acute brain slice electrophysiology in 14-week-old mice, we discovered that exposure to supraphysiological levels of O_2_ during the first two postnatal weeks leads to heightened presynaptic excitability, increased strength of basal synaptic transmission, and a greater magnitude of LTP at CA3–CA1 synapses. Previously, we reported deficits in spatial memory and increased anxiety in this model (Ramani et al., 2013), findings that mimic neurobehavioral deficits seen in adolescents born preterm who received prolonged periods of supplemental O_2_ in the neonatal intensive care unit. In addition to neurobehavioral deficits, preterm infants are also at high risk for clinical and subclinical seizures compared to term-born infants (22.2% compared to 0.5%; Scher, 2006). Therefore, the abnormalities in synaptic transmission and heightened excitability we observe in this model suggest similar changes could be a major contributor to the cognitive and neurobehavioral deficits and seizures seen in the adolescents born preterm.

The pathophysiological processes by which early life hyperoxia exposure increases CA3–CA1 synapse excitability are likely complex. In this study, early life hyperoxia exposure leads to a larger steady-state postsynaptic dendritic depolarization later in life, which suggests that the larger magnitude of LTP we observe at CA3–CA1 synapses in the hyperoxia-exposed mice might be due to the heightened postsynaptic dendritic depolarization. Further, the increased basal synaptic strength and heightened axon excitability revealed in the I/O curves, in addition to the increase in CA1 dendritic excitability observed in the CBI analysis in the neonatal hyperoxia-exposed young adult mice, indicates that early life O_2_ exposure also markedly enhances excitability in the CA3–CA1 circuit later in life. The increased fiber volley amplitudes at CA3–CA1 synapses we observed indicate either a decrease in action potential threshold in the axons or that a larger number of axons are recruited by electrical stimulation during the generation of the I/O curves and LTP induction. The lack of change in PPR suggests there is no change in the presynaptic release probability of glutamate, indicating that the heightened responses are not likely caused by abnormal function of axon terminals. Previously, we reported that hyperoxia reduces the expression of hippocampal potassium voltage-gated channel subfamily D member 2 (KCND2) protein at 14 weeks (Ramani et al., 2018). Therefore the action potential threshold may be decreased as a consequence of neonatal hyperoxia. Future studies are needed to determine how hyperoxia conditions during the neonatal period induce altered excitability and whether axon sprouting occurs that remains in adulthood. Further investigations examining other stimulation protocols, such as high-frequency stimulation, the LTP magnitude at saturation following several rounds of stimulation, and long-term depression, will also provide additional mechanistic information in our model.

Another important consideration is the possibility of decreased GABAergic inhibition, as inhibitory interneurons play a critical role in maintaining the excitatory and inhibitory balance. Since developing GABAergic interneurons are highly vulnerable to oxidative stress (Cabungcal et al., 2013), likely, hyperoxia-exposed young-adult mice may also have deficits in GABAergic function. Thus, weakened inhibition would be expected to facilitate the induction of LTP, leading to a heightened potentiation, and it could also be responsible for the CA1 dendritic hyperexcitability we observed in our neonatal hyperoxia model. In future studies, detailed investigations are needed into a potential loss of GABAergic interneurons leading to decreased strength of inhibitory transmission and increased excitation/inhibition balance. It is also possible that hyperoxia exposure compromises the health of GABAergic interneurons and GABAergic tone resulting in CA3–CA1 synaptic hyperexcitability. Future electrophysiological experiments recording from GABAergic interneurons and recording in GABA_A_ receptor blockers (e.g., Picrotoxin, Bicuculline) may help determine the role of GABAergic tone on hyperoxia-induced CA3–CA1 hyperexcitability we observed in this neonatal hyperoxia model. In future studies, we will also employ whole-cell current-clamp studies to investigate at the single-cell level precise changes in intrinsic membrane properties including resting membrane potential, input resistance, and action potential threshold, and will use voltage-clamp to gain a more detailed understanding of alterations in synaptic transmission, including the excitation/inhibition balance, and possible deficits in GABAergic inhibition.

Our study used TBS to induce LTP (Larson et al., 1986) instead of high-frequency stimulation. TBS mimics the complex-spike discharges of the pyramidal neurons (Rudell et al., 1980; Rudell and Fox, 1984) that occur spontaneously during learning and memory formation. The optimal repetition rate used in TBS corresponds to the frequency of the hippocampal theta rhythm, an EEG pattern related to memory storage processes (Vanderwolf, 1969). Though this study assessed the changes in LTP magnitude at CA3–CA1 synapses induced by early-life OS, we did not investigate possible changes in LTP induction or expression at other hippocampal synapses such as the perforant pathway to the dentate gyrus or to distal CA1 dendrites (temporammonic pathway), which provide a major input to the hippocampus from the entorhinal cortex and is involved in memory consolidation. Also, while experiments are designed to evaluate the effect of early life hyperoxia exposure on hippocampal development, hyperoxia-induced physiological changes in the dams might have adversely impacted hippocampal development in hyperoxia exposed mice pups.

Our findings are consistent with neonatal mouse models of ischemia and reperfusion injury (Zanelli et al., 2015) but are in contrast with many models of OS-associated brain disorders, which linked OS-induced LTP deficits to the cognitive decline (Bisht et al., 2018; Matikainen-Ankney et al., 2018; Smith and McMahon, 2018a). This indicates that OS affects the developing brain differently than the adult brain and leads to long-lasting impairment in synaptic function. Additional electrophysiological studies are needed to assess the excitatory and inhibitory balance, action potential generation, interneuron function, axonal sprouting, and dendritic arborization. A better understanding of these systems is required to determine the mechanisms by which early life OS leads to long-lasting synaptic dysfunction.

## Data Availability Statement

The raw data supporting the conclusions of this article will be made available by the authors, without undue reservation.

## Ethics Statement

The animal study was reviewed and approved by UAB Institutional Animal Care and Use Committee (IACUC).

## Author Contributions

MR and LM: concept and design. KM: electrophysiology. MR, NA, KM, and LM: data analysis and interpretation, and drafting the manuscript for important intellectual content. All authors contributed to the article and approved the submitted version.

## Conflict of Interest

The authors declare that the research was conducted in the absence of any commercial or financial relationships that could be construed as a potential conflict of interest.
